# Assessing environmental and climatic predictors of dengue fever in Santa Marta, Colombia: implications for One Health surveillance

**DOI:** 10.1016/j.soh.2026.100164

**Published:** 2026-06-06

**Authors:** Mia E. Martin, Juan A. Insaurralde, Francisco F. Ludueña-Almeida, Doriam Camacho-Rodríguez, Gabriel Parra-Henao, Alexander Salazar-Ceballos, Elizabet L. Estallo

**Affiliations:** a Universidad Nacional de Córdoba, Facultad de Ciencias Exactas, Físicas y Naturales, Centro de Investigaciones Entomológicas de Córdoba, Córdoba, X5000HUA, Argentina; b Instituto de Investigaciones Biológicas y Tecnológicas, Consejo Nacional de Investigaciones Científicas y Técnicas, Universidad Nacional de Córdoba, Córdoba, X5000HUA, Argentina; c Universidad Nacional de Córdoba, Facultad de Ciencias Exactas, Físicas y Naturales, Departamento de Matemática, Córdoba, X5000HUA, Argentina; d Universidad Cooperativa de Colombia, Campus Santa Marta, Facultad de Enfermería, Santa Marta, 470004, Colombia; e Centro de Investigación en Salud para el Trópico, Universidad Cooperativa de Colombia, Campus Santa Marta, Facultad de Medicina, Santa Marta, 470004, Colombia

**Keywords:** Dengue, One Health, Epidemiology, Climate change, Environmental determinants, Vector-borne disease, Colombia, Urban health, Prevention strategy

## Abstract

**Background:**

Dengue fever is a major mosquito-borne disease whose transmission is influenced by climatic, environmental, and demographic factors. Colombia is a hyperendemic country, yet long-term local-scale studies remain limited. This study assessed the relationships between dengue incidence and environmental, climatic, and demographic predictors in Santa Marta, Colombia.

**Methods:**

Weekly dengue case counts from 2009 to 2023 were analyzed using generalized linear mixed models with a negative binomial distribution. Epidemiological data were integrated with remotely sensed environmental variables, including vegetation indices (Normalized Difference Vegetation Index [NDVI] and Normalized Difference Water Index [NDWI]), daytime and nighttime Land Surface Temperature (LST), precipitation, and human population density. Lagged environmental effects (1–4 weeks) and biologically plausible interactions were evaluated.

**Results:**

A total of 9237 dengue cases were recorded during the study period. The best-fitting model identified significant positive associations between dengue incidence and NDVI (*P* < 0.001). Significant negative associations were observed for maximum daytime LST at a one-week lag (*P* < 0.001), minimum nighttime LST at a four-week lag (*P* < 0.001), NDWI at a one-week lag (*P* = 0.018), and precipitation at a four-week lag (*P* < 0.001). Population density significantly strengthened the positive effect of NDVI on dengue cases.

**Conclusions:**

Dengue transmission in Santa Marta is shaped by complex and delayed interactions among environmental, climatic, and demographic factors. Vegetation cover increased dengue risk, particularly in densely populated areas, whereas excessive rainfall and high temperatures were associated with reduced incidence. These findings support the development of One Health-based surveillance and early-warning systems for dengue prevention.

## Graphical abstract

**Figure:**
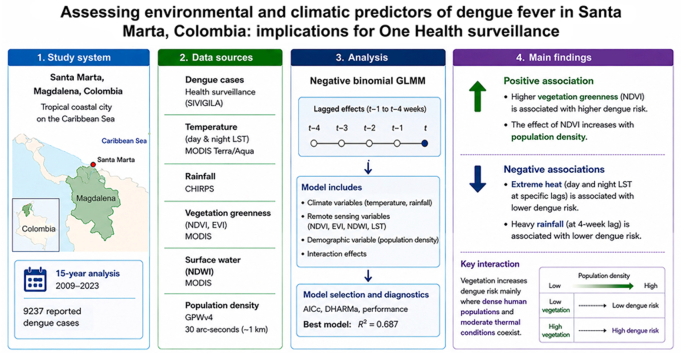

## 1 Introduction

Accelerated climate change, driven by global warming, poses a significant risk to public health. In recent years, there has been a continuous increase in global temperatures, along with a rise in the frequency of extreme weather events, which impact health both directly and indirectly [[1], [2], [3], [4]]. According to the World Meteorological Organization (WMO), 2024 was likely the first calendar year in which the global mean surface temperature exceeded 1.5 °C above the 1850–1900 pre-industrial average, reaching approximately (1.55 ± 0.13) °C [5]. This continuous rise in global temperature has contributed to the increased frequency and spread of climate-sensitive vector-borne diseases, such as dengue fever [4]. The WHO reported approximately 505,000 dengue cases globally in the year 2000, whereas by 2024, more than 14.6 million cases had been reported [1], highlighting a continuous increase in dengue cases parallel to the rise in global temperature [3,4].

Dengue fever is currently the most important arboviral disease affecting humans, with more than half of the world's population at risk of infection [1]. It is transmitted mainly by *Aedes aegypti* and, to a lesser extent, by *Ae.* *albopictus*, two mosquito species highly sensitive to climatic and environmental conditions [6]. In particular, precipitation, temperature, and humidity strongly influence vector survival, reproduction, and viral replication [7,8]. These factors also correlate with the incidence of dengue each year in tropical and subtropical climates in complex, nonlinear ways that vary in space and time, often with delays ranging from several weeks to months [9,10]. Specifically, dengue virus transmission increases with rising temperatures, peaking at 26–29 °C in mean temperatures, and then declines above this thermal optimum [11]. Rainfall causes water to accumulate in containers and infrastructure in urban environments, creating an aquatic habitat for the development of pre-imaginal mosquitoes. Human water storage practices further complicate this dynamic, as people living in water-scarce regions (or in settlements with precarious access to water) store water in containers distributed throughout the home and neighborhood, which serve as egg-laying sites for female mosquitoes. In addition to climatic determinants, urbanization, inadequate water management, and population growth contribute to the expansion of dengue transmission [12,13].

Latin America has emerged as a region with a high dengue burden, reporting recurrent and increasingly severe outbreaks over the last two decades [14]. Within the region, Colombia is a hyperendemic country where all four dengue serotypes co-circulate [15]. The country has reported cyclical dengue epidemics, with at least five major outbreaks between 1971 and 2020 (1998, 2002, 2010, 2013, and 2019) [14]. Dengue transmission in Colombia is shaped not only by favorable climatic conditions but also by socio-demographic and environmental factors, such as rapid urban growth, socioeconomic inequalities, and limited access to public health services [6,14].

Santa Marta, a coastal city in Northern Colombia, represents a particularly vulnerable setting for dengue transmission due to its tropical climate, high human population density, and environmental characteristics. Despite being an endemic area, we did not identify previous studies examining dengue–climate–environment relationships in Santa Marta specifically. Most research linking climatic, environmental, and demographic factors to dengue risk in Colombia has been conducted in other urban centers, particularly in Apulo, Anapoima, La Mesa, Cali, Medellín, and Girardot. Studies in these cities have demonstrated the influence of temperature variation and local weather on dengue transmission dynamics [[16], [17], [18]], as well as spatial patterns and neighborhood-level socio-environmental determinants of transmission [[19], [20], [21]]. Additional work has explored space-time clustering of dengue and co-circulation with other arboviral diseases [21], and fine-scale spatial distributions in hyper-endemic contexts [22]. Collectively, these studies highlight the relevance of localized analyses for understanding dengue dynamics in Colombia, while underscoring the existing knowledge gap for cities such as Santa Marta.

The present study aims to analyze more than a decade of dengue cases in Santa Marta, Colombia (2009–2023), exploring their relationship with meteorological, environmental, and demographic determinants. By integrating epidemiological and environmental data, this study contributes to a One Health framework that acknowledges the interconnection between human health, mosquito ecology, and environmental change. Such an approach is essential to strengthen prevention and control strategies in Colombia and the broader Latin American region, particularly in the face of increasing climate variability and urban expansion.

## 2 Materials and methods

### 2.1 Study area

**Fig. 1:**
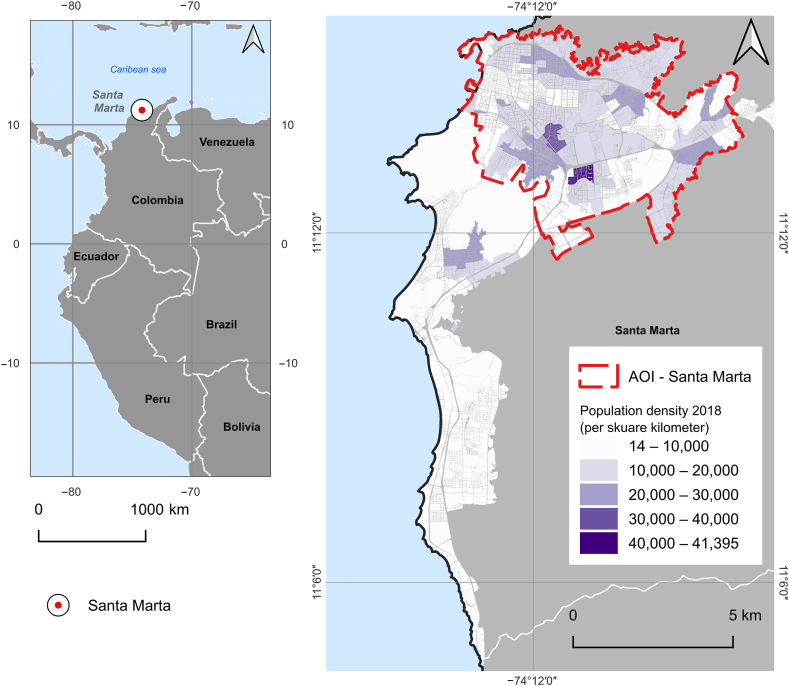
The city of Santa Marta with the population density and the area of interest (AOI) as the study area. The city is located in the Department of Magdalena, Municipality of the same name, Colombia. The map was created by the authors using QGIS Desktop (version 3.44) based on population density data 2018 from the Departamento Administrativo Nacional de Estadística (https://www.dane.gov.co/) and administrative boundary layers from publicly available geographic databases.

Santa Marta city (11.241° N, 74.199° W) is located in the Magdalena Department of Colombia and covers a total area of 55.10 km^2^, with an estimated population of 548,202 inhabitants (https://www.santamarta.gov.co/geografia). The study area corresponded to the area of interest (AOI) and was limited to a smaller portion of the total city area, with a total area of 37.03 km^2^. It was deliberately defined by prioritizing sectors with the highest population density and by applying a spatial delineation that differentiates permanent residential areas from non-permanent (tourist-oriented) residential zones (Fig. 1). Permanent residential areas were defined as the AOI because they represent the population at risk for dengue and are the areas where cases are consistently captured by official public health surveillance systems. In contrast, non-permanent residential areas, predominantly located in the southern portion of the city, were excluded because visitors are not routinely registered in local surveillance data and dengue cases may be diagnosed after individuals return to their place of residence, leading to underreporting and potential misclassification. This spatial criterion allowed for a more accurate alignment between environmental exposure, resident population, and reported dengue cases.

The city is located in the bay of the same name on the Caribbean Sea and surrounded by the Sierra Nevada de Santa Marta. The average altitude of the city is 2 m.a.s.l., with an altitudinal gradient from sea level to 5775 m.a.s.l. at Cristóbal Colón peak (68 km southeast in a straight line), which is the highest peak in Colombia, located in the Sierra Nevada de Santa Marta [23]. According to the climatic classification of Köppen-Geiger, Santa Marta is characterized by a tropical savanna climate (Aw), bordering on a hot semi-arid climate, with largely uniform temperatures throughout the year [24]. This pronounced topographic contrast influences local temperature, humidity, and drainage patterns, which in turn affect the availability and persistence of breeding sites for *Aedes* mosquitoes. The dry season runs from December to April, with February being the driest month (3 mm), and the total rainfall for the dry season is 76 mm [25]. On the other hand, the wet season extends from May to November, with May being the rainiest month, recording an average monthly precipitation of 339 mm. Seasonal fluctuations in rainfall are ecologically important, as they regulate the formation of both natural and artificial water-holding containers, thereby shaping the temporal dynamics of mosquito abundance and dengue transmission risk.

Due to climate change, in the past 10 years, Santa Marta is a city that experiences long periods of drought lasting up to 10 months without rain. It also has an intermittent water supply that does not reach the entire city. Because of this, citizens have elevated tanks, low tanks, and all kinds of jars and pots in which they store water. These containers, mainly low tanks and jars and pots, are the main breeding sites for *Ae. aegypti* in this city.

The average annual temperature is 25.4 °C, with minimums of around 24 °C and maximums of approximately 33 °C. The annual average thermal amplitude is 3.5 °C with a maximum in March (3.9 °C) and a minimum in June and December (3.1 °C). The mean annual precipitation is 977 mm, being relatively moderate. Humidity is 75% or higher throughout the year, and the number of sunny days decreases gradually from January to October [25].

### 2.2 Data collection

#### 2.2.1 Dengue cases data

The dengue cases data were obtained from the “Microdata Search” system of “Sistema Nacional de Vigilancia en Salud Pública” (Public Health Surveillance System, SIVIGILA, available at https://portalsivigila.ins.gov.co/), managed by the “Instituto Nacional de Salud” (National Institute of Health). This platform provides curated, anonymized databases of public health events dating back to 2007, with a spatial resolution at the city level. After selecting the “dengue” event and specifying the study years (2009 to the 2022–2023 outbreak season), the data were downloaded to the SIVIGILA web 4.0 system. Daily dengue cases data were aggregated by their corresponding epidemiological week, facilitating temporal analysis.

#### 2.2.2 Demographic data

As a source of demographic data, we used the Gridded Population of the World, Version 4 (GPWv4): Population Density, Revision 11 [26]. This dataset provides estimates of human population density (individuals/km^2^) derived from national census and population register data for the years 2000, 2005, 2010, 2015, and 2020, with a spatial resolution of 30 arc-seconds (∼1 km at the equator). Population density rasters were generated by dividing the population count raster for each reference year by the corresponding study area (AOI) raster.

To process these data, we developed a workflow in R (version 4.2.3) to extract population density values for Santa Marta city, Colombia. This involved cropping the rasters to the AOI, masking non-relevant regions, and extracting mean population density values within the target boundaries. Temporal interpolation and extrapolation were applied using polynomial regression to estimate values for the years 2009–2023, ensuring consistency across the study period.

#### 2.2.3 Meteorological and environmental data

Meteorological data were obtained from the Climate Hazards Center InfraRed Precipitation with Station data (CHIRPS) [27] quasi-global dataset corresponding to each epidemiological week in Santa Marta, Colombia. The CHIRPS data have a spatial resolution of approximately 0.05° (5600 m) and provide daily information. Environmental data were obtained from the MODIS sensors Terra and Aqua for the AOI, with a frequency that coincides with the epidemiological weeks of the study period. The spatial resolution of these variables corresponds to 1000 m, and the temporal resolution is 1 day. The Moderate Resolution Imaging Spectroradiometer (MODIS) images were preprocessed to remove them from the statistics calculation by applying a cloud mask. From these sensors and data sets, the maximum value, mean, median, minimum, mode, standard deviation, sum, and variance were calculated for each epidemiological week from 2009 to 2023 for the AOI. For the MODIS sensor data, the weekly median was estimated, and then the aforementioned zonal statistics were calculated. For the CHIRPS data, the weekly precipitation sum was estimated, and then the zonal statistics were calculated. This process was performed using Google Colab (available at https://colab.research.google.com) and Google Earth Engine [28]. The variables calculated were Normalized Difference Vegetation Index (NDVI), Enhanced Vegetation Index (EVI), Land Surface Temperature during the day (LSTD) and night (LSTN), Normalized Difference Water Index (NDWI), and precipitation (P), which were selected due to their relevance in dengue cases and mosquito vector studies according to the bibliography [[29], [30], [31]]. The data were obtained using Google Earth Engine and processed using Python (version 3.10).

### 2.3 Data analysis

We employed a generalized linear mixed model (GLMM) to examine the association between meteorological, environmental and demographic covariates and dengue case counts over the study period. The analysis focused on estimating long–term relationships between dengue cases and explanatory variables. GLMMs are well-suited for this purpose, as they allow for flexible distributional assumptions for count data and the inclusion of random effects to account for unobserved heterogeneity.

Temporal dependence and seasonality are well-recognized features of dengue transmission. In this study, temporal structure was partially addressed through the inclusion of time-varying climatic covariates that capture seasonal and interannual variability in predicted variables. Additionally, seasonality in dengue transmission was explicitly evaluated by testing alternative temporal structures in the modeling framework. “Week” was initially included as a random effect to capture recurrent within-year seasonal variation. In addition, “year” was tested as a random effect to account for interannual variability, and long-term trends in dengue cases. Model comparisons indicated that including year as a random effect provided a better fit to the data than modeling week as a random effect. Consequently, “year” was retained in the final model specification. Seasonal patterns were further captured indirectly through time-varying climatic covariates, which reflect both intra-annual and interannual environmental variability relevant to mosquito dynamics and virus transmission.

The weekly number of reported dengue cases for epidemiological week *t* from 2009 to 2023 (DC_*t*_) was used as the response variable. Explanatory variables describing meteorological, environmental, and demographic conditions, NDVI, EVI, LSTD, LSTN, NDWI, precipitation (P), human population density (PD) and week (w) as a random factor. Each meteorological and environmental variable was analyzed using its raw, mean, maximum, and minimum values, as well as with time lags of 1, 2, 3, and 4 weeks to capture potential delayed effects in the dengue cases [32]. Thus, *M*LSTD_*t*−1_ corresponds to the maximum LTSD recorded in the week immediately preceding the week of the reported dengue cases (lag 1). Similarly, *m*LSTN_*t*−4_ represents the minimum LSTN values recorded at four-week lags. In both cases, the lag refers to a specific week (*t*−1, *t*−2, *t*−3, or *t*−4) and does not involve aggregation across multiple weeks. Furthermore, potential interaction effects between the explanatory variables were explored based on biological plausibility, particularly the interactions between meteorological variables and population density.

First, data exploration was conducted according to the protocol outlined by Zuur et al. [33]. The explanatory variables were standardized to balance their weights and avoid introducing errors in the model due to the different measurement units of each variable. Following this, and to avoid multicollinearity, Spearman's correlation test was computed for all pairs of independent variables and also with the response variable. A threshold of |*r*| < 0.7 was used to retain only non-collinear variables. Variables with stronger correlations were systematically removed. For each environmental variable with multiple lagged versions, the Spearman's correlation coefficient with DC_*t*_ was calculated. The lag exhibiting the highest absolute value correlation (|*r*|) was selected as the representative predictor for that variable. This step ensured that only the most relevant time lag was included in the final model.

After the data analysis and given the count nature of the response variable, initial models were fitted assuming a Poisson error distribution. Model diagnostics indicated substantial overdispersion, and alternative specifications using the negative binomial distribution were subsequently evaluated. The negative binomial model provided a better fit to the data, as evidenced by improved goodness-of-fit and reduced residual overdispersion, and was therefore retained for inference. All models used a log link function [34].

To identify the most parsimonious model explaining the variation in DC_*t*_, we performed a stepwise model selection procedure using Akaike's information criterion (AIC). First, we fitted a full model using all the non-correlated independent variables (Table 1). Then, to optimize model selection, we used the “dredge” function from the “MuMIn” package [35], which systematically evaluated all possible model subsets based on AIC values. The model with the lowest AIC (with a difference of at least two points) was selected as the best-fitting model, balancing goodness-of-fit with complexity. After obtaining the best model, multicollinearity among predictors was assessed using the variance inflation factor (VIF), ensuring that all included variables had VIF values < 5. To validate the best model, the coefficient of determination (*R*^2^) was computed to assess the extent to which the observed values align with those estimated by the model. Additionally, the model's predictive performance and zero-inflation test were evaluated using “performance” [36] and “DHARMa” [37] packages.

**Table 1:** Explanatory variables selected and included in the model construction after the data analysis.

Variable name	Definition
PD	Human population density as individuals per square kilometer
NDVI_*t*_	Average normalized difference vegetation index obtained for the week when dengue cases were reported
*M*LSTN_*t*_	Maximum land surface temperature during the night obtained for the week when dengue cases were reported
*M*LSTD_*t*−1_	Maximum land surface temperature during the day obtained for the previous week (lag 1) in which dengue cases were reported
*s*LSTD_*t*−3_	Standard deviation of land surface temperature during the day obtained for the previous three weeks (lag 3) in which dengue cases were reported
*m*LSTN_*t*−4_	Minimum land surface temperature during the night obtained for the previous four weeks (lag 4) in which dengue cases were reported.
NDWI_*t*−1_	Normalized difference water index mode obtained for the previous week (lag 1), in which dengue cases were reported
P_*t*−4_	Average accumulated precipitation obtained at four-week lags (lag 4) in which dengue cases were reported

Note: *t* represents the time lag (in weeks) between the environmental variable and the reported dengue cases.

Because dengue transmission exhibits strong temporal structure, residual diagnostics were conducted to assess remaining temporal dependence after accounting for covariates. Simulation-based residuals were generated using “DHARMa” package, and residual time series and autocorrelation function (ACF) plots were examined. Residuals were also aggregated by time period to evaluate temporal autocorrelation at the population level. These diagnostics indicated remaining temporal autocorrelation, suggesting that short-term dependence and transmission persistence were not fully captured by the GLMM framework.

Accordingly, model results are interpreted as estimates of long-term associations between DEN cases and climatic, environmental, and demographic drivers rather than as explicit representations of short-term transmission dynamics. More complex autoregressive or fully spatiotemporal time-series approaches are acknowledged as valuable extensions for future work.

All statistical analyses were conducted using R software (version 4.2.3). The scripts in both R and Python software will be available in a GitHub repository for consultation (https://github.com/miemartin/DEN-santa-marta/).

## 3 Results

### 3.1 Descriptive statistics

#### 3.1.1 Dengue cases data

**Fig. 2:**
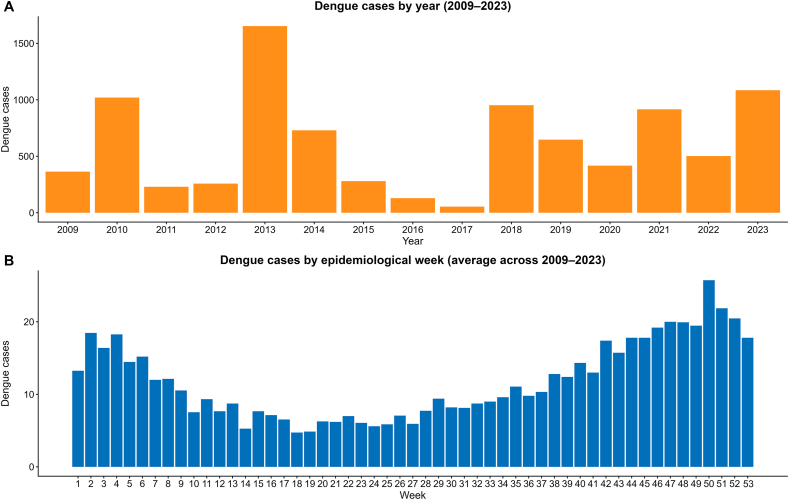
Temporal distribution of dengue cases in Santa Marta. A: Annual case counts from 2009 to 2023; B: Average number of cases per epidemiological week across all study years.

The dataset included 9237 observations of dengue cases over 15 years. Fig. 2A shows the annual variation in reported dengue cases from 2009 to 2023. The highest number of cases was recorded in 2013, exceeding 1600 cases. Other peak years include 2010, 2018, 2021 and 2023, with over 800 reported cases. In contrast, the lowest dengue number was observed in 2017, with fewer than 100 cases. The data exhibit fluctuations over time, with periodic increases and decreases in reported dengue cases.

Fig. 2B illustrates the distribution of dengue cases across epidemiological weeks over several years (2009–2023). The seasonal pattern reveals a bimodal distribution, with higher case numbers at the beginning and end of the year. Dengue cases are relatively high between weeks 1–15, followed by a decline between weeks 16–35. A subsequent increase is observed from week 36 onward, peaking around weeks 49–53.

#### 3.1.2 Climatic, environmental, and demographic data

Descriptive analysis of independent variables showed that the population density ranged from 237.75 to 337.02 inhabitants per square kilometer. The mean NDVI ranged from 0 to 0.554, with an average of 0.363, while the minimum NDVI varied between −0.003 and 0.275, with a mean of 0.147. Maximum LSTD ranged from 0 °C to 44.71 °C, with a mean of 37.35 °C, while the standard deviation of LSTD varied from 0 °C to 4.56 °C, with an average of 1.72 °C. Minimum LSTN ranged from −2.75 °C to 28.53 °C, with a mean of 23.01 °C. The mode of NDWI ranged from −0.724 to 0, with an average of −0.384. Regarding precipitation, mean values ranged from 0 mm to 49.732 mm, with an average of 4.059 mm, while minimum precipitation varied between 0 mm and 2.023 mm, with a mean of 0.158 mm.

**Fig. 3:**
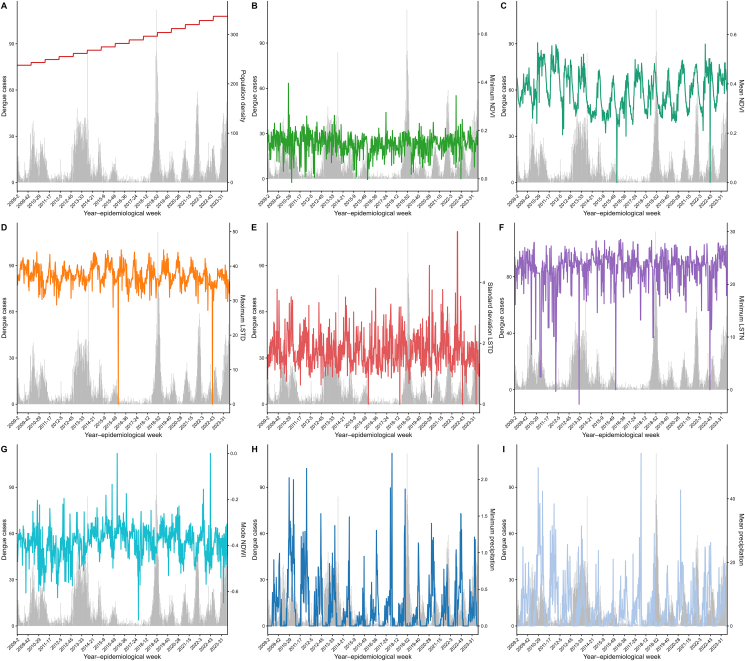
Temporal distribution of dengue cases and key independent variables. Weekly dengue case counts (gray) are plotted against population density (A), minimum NDVI (B), mean NDVI (C), maximum LSTD (D), standard deviation of LSTD (E), minimum LSTN (F), mode NDWI (G), minimum precipitation (H), and mean precipitation (I). Abbreviations: NDVI, Normalized Difference Vegetation Index; LSTD, Land Surface Temperature during the day; LSTN, Land Surface Temperature during the night; NDWI, Normalized Difference Water Index.

The time-series analysis (Fig. 3) illustrates the temporal relationship between dengue cases and key independent variables. The observed values of population density increase until the most recent year (Fig. 3A), reflecting a progressive increase in urbanization and human concentration. The NDVI plots (Fig. 3B and C) reveal seasonal trends, where higher NDVI values (indicating greater vegetation coverage) often align with increased dengue numbers. Similarly, LST trends (Fig. 3D–F) show that both maximum LSTD and minimum LSTN exhibit fluctuations, with higher temperatures occasionally coinciding with dengue outbreaks. The NDWI plot (Fig. 3G) indicates predominantly negative values, suggesting dry conditions, while precipitation peaks (Fig. 3H and I) appear to precede increases in dengue cases, highlighting a possible lag effect between rainfall and dengue cases.

### 3.2 Best model selection and performance

The best GLMM was selected based on the lowest AIC values (Table S1). The model had an AIC value of 4928.05 and an *R*^2^ value of 0.687. In contrast, the null model had a higher AIC of 5084 and a lower *R*^2^ of 0.521. The systematic component of the best model was specified as follows:

DC_*t*_ = 2.136 + 0.058PD + 0.126NDVI_*t*_ – 0.228*M*LSTD_*t*–1_ – 0.080*m*LSTN_*t*–4_ – 0.086NDWI_*t*–1_ – 0.236P_*t*–4_

The estimated effects from the best model (Table 2) suggest that the mean values of NDVI_*t*_ were positively associated with dengue cases (DC_*t*_). In contrast, other variables with time lags, such as *M*LSTD_*t*–1_, *m*LSTN_*t*–4_, NDWI _*t*–1_, and P_*t*–4_, all exhibited significant negative relationships with dengue cases DC_*t*_. Additionally, significant positive interactions between NDVI_*t*_ with PD, and between NDVI_*t*_ with *M*LSTD_*t*–1_ have been shown.

**Table 2:** Coefficients of the best generalized linear mixed model (GLMM).

Variable	Estimate	Standard error	*z* value	*P*
PD	0.058	0.225	0.256	0.800
NDVI_*t*_	0.126	0.040	3.110	**<0.001**
*M*LSTD_*t*–1_	−0.228	0.038	−5.925	**<0.001**
*m*LSTN_*t*–4_	−0.080	0.031	−2.599	**<0.001**
NDWI_*t*–1_	−0.086	0.036	−2.358	0.018
P_*t*–4_	−0.236	0.032	−7.436	**<0.001**
PD × NDVI_*t*_	0.122	0.031	3.899	**<0.001**
NDVI_*t*_ × *M*LSTD_*t*–1_	0.110	0.030	3.824	**<0.001**

Note: *t* represents the time lag (in weeks) between the environmental variable and the reported dengue cases.

Abbreviations: PD, population density; NDVI, Normalized Difference Vegetation Index; *M*LSTD, maximum Land Surface Temperature during the day; *m*LSTN, minimum Land Surface Temperature during the night; NDWI, Normalized Difference Water Index; P, precipitation.

**Fig. 4:**
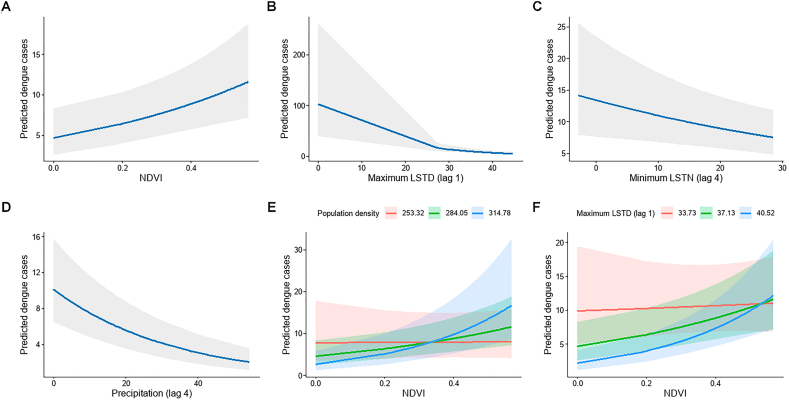
Predicted dengue cases (DC_*t*_) derived from the final negative binomial mixed-effects model with a log link function. A–D: The marginal effects of the Normalized Difference Vegetation Index (NDVI_*t*_) (A), maximum LSTD at a one-week lag (*M*LSTD_*t*–1_) (B), minimum LSTN for the previous four weeks (*m*LSTN_*t*–4_) (C), and precipitation for the previous four weeks (P_*t*–4_) (D). E–F: Interaction effects between NDVI_*t*_ and population density, and between NDVI_*t*_ and *M*LSTD_*t*–1_. Colored curves represent low (red), intermediate (green), and high (blue) values of the interacting variable included in the interaction term. Shaded areas represent 95% confidence intervals. Abbreviations: DC, dengue case; NDVI, Normalized Difference Vegetation Index; LSTD, Land Surface Temperature during the day; LSTN, Land Surface Temperature during the night.

Fig. 4 summarizes the marginal effects of environmental and climatic predictors on the predicted number of dengue cases (DC_*t*_). Overall, non-linear relationships were observed, with clear differences in magnitude and direction among predictors and interaction effects. Predicted dengue cases DC_*t*_ increased with NDVI_*t*_ (Fig. 4A), indicating higher dengue numbers in areas with higher NDVI values. In contrast, maximum LSTD (*M*LSTD_*t*–1_) showed a strong negative association with dengue cases DC_*t*_ (Fig. 4B): predicted cases declined sharply as maximum LSTD increased, approaching minimal values at the highest temperatures. Similarly, minimum LSTN (*m*LSTN_*t*–4_) was negatively associated with dengue cases DC_*t*_ (Fig. 4C), with higher predicted cases at lower minimum temperatures. Precipitation at four-week lags (P_*t*–4_) also exhibited a decreasing, nonlinear effect (Fig. 4D). Predicted dengue cases were highest at low precipitation levels and declined steadily as precipitation increased, suggesting a limiting effect of excessive rainfall on dengue transmission after the specified lag.

Interaction effects revealed marked modulation by population density and temperature. The relationship between NDVI_*t*_ and dengue cases DC_*t*_ varied by population density (Fig. 4E): at high population density, predicted cases increased steeply with NDVI, whereas at low population density, the effect of NDVI was weak and nearly flat. Likewise, the effect of NDVI_*t*_ depended on *M*LSTD_*t*–1_ (Fig. 4F). Under higher LSTD max conditions, predicted dengue cases rose sharply with increasing NDVI, while under lower maximum LSTD conditions, the NDVI effect was modest.

## 4 Discussion

This study presents a comprehensive long-term analysis of dengue dynamics in Santa Marta, Colombia, linking epidemiological trends with meteorological, environmental and demographic determinants from 2009 to 2023. Our results reveal that vegetation indices (NDVI) were positively associated with dengue cases, whereas LST (both day and night, at specific lags) and precipitation showed significant negative associations. These results are consistent with studies conducted in other Colombian cities, such as Cali, Medellín, and Girardot, where meteorological and landscape variables have similarly shaped dengue transmission dynamics [16,[18], [19], [20], [21]]. Importantly, our findings show that the relationship between vegetation and dengue transmission is strongly modulated by population density and LSTD, indicating that environmental suitability alone is not sufficient to drive transmission unless accompanied by sustained human exposure. These interaction effects highlight the context-dependent nature of dengue risk and reinforce the need for geographically resolved analyses when studying environmental determinants of arboviral diseases.

Vegetation indices, particularly NDVI, exhibited a positive relationship with dengue cases in the best model. This relationship suggests that areas with higher NDVI values may favor mosquito proliferation, likely through the availability of shaded and humid microhabitats suitable for *Aedes* breeding, especially in urban settings with unmanaged green spaces. NDVI reflects the combined effect of vegetation quantity and quality, rather than distinguishing specific vegetation types or land-use structures [38]. Consequently, higher NDVI values in urban environments may represent heterogeneous landscape elements such as trees, mixed vegetation, unmanaged vacant lots, or peri-domestic green areas. Similar associations have been reported in other Colombian regions, where vegetation, NDVI values, and peri-urban environments enhance vector survival and human–mosquito contact [39,40]. Picinini Freitas et al. [39] also indicate that dengue was more prevalent in areas with higher NDVI, which may reflect increased transmission in vegetated peri-urban or heterogeneous urban environments rather than a strict ruralization process. This trend could be related to the maximum vector and virus tolerance temperature threshold. In low-vegetated cities, heatwaves are generated, negatively affecting the vector development and dengue infections if the temperatures rise above the maximum threshold, while in vegetated cities, the conditions would be more appropriate [41].

In our study, NDVI was used as a proxy for greenness, capturing the combined effect of vegetation quantity and quality; however, this metric does not distinguish between different vegetation types (e.g., arboreal cover, managed urban parks, or unmanaged grassy vacant lots), which may have contrasting implications for *Ae. aegypti* ecology. Evidence from Brazilian cities suggests that the relationship between vegetation and dengue is highly context-dependent. For instance, Cunha et al. [38] showed that higher vegetation indices associated with tree cover were linked to lower dengue risk, likely reflecting better environmental quality and reduced availability of artificial breeding sites. In contrast, other studies in Brazil have reported that poorly managed green areas or vacant lots dominated by herbaceous vegetation may favor mosquito proliferation due to the accumulation of containers and limited urban sanitation [42,43]. These contrasting findings are consistent with the well-established ecology of *Ae*. *aegypti* as a highly anthropophilic and urban-adapted species, with limited capacity to persist in preserved natural or densely vegetated areas [44].

Furthermore, other studies in Colombia demonstrate that dengue incidence can be modulated by the spatial heterogeneity of green cover, urban growth, and socioeconomic factors [45,46]. This interpretation is reinforced by the interaction observed between NDVI and population density, where vegetation greenness was associated with a marked increase in dengue cases only under high population density conditions. This positive association should be interpreted within the context of urban vegetation heterogeneity. In tropical cities, higher NDVI values do not necessarily indicate dense natural forest but may also reflect peri-domestic vegetation, vacant lots, unmanaged green areas, or mixed urban vegetation that can accumulate artificial containers and other potential breeding sites for *Ae. aegypti* [38]. Vegetated urban spaces may indirectly facilitate mosquito persistence by providing shaded and thermally microhabitats that improve adult survival and breeding-site stability [44]. In our study, the effect of NDVI was stronger in areas with higher population density, suggesting that vegetation may increase dengue risk primarily in densely populated urban settings, where the availability of human hosts and artificial breeding sites facilitates transmission [38].

In addition, precipitation showed a negative effect at lagged periods, indicating that lower rainfall several weeks prior was associated with higher dengue incidence. At a national scale in Colombia (2007–2017), precipitation was found to be negatively correlated with the number of dengue cases [47]. While precipitation is generally considered a driver of mosquito breeding, excessive or intense rainfall can wash away larvae from breeding sites and reduce vector abundance, followed by a rebound in transmission during subsequent drier weeks [3,40]. This pattern was evident in Santa Marta, where lower precipitation at four-week lags aligned with increased dengue cases. Similar lagged and nonlinear effects have been reported in Caribbean, Colombian and Brazilian regions, underscoring the need for flexible models that capture delayed climate impacts. Consistent with our findings, Lowe et al. [9] demonstrated in Barbados that drought conditions were associated with increased dengue risk at a 3- to 5-month lag, whereas heavy rainfall and high minimum temperatures elevated risk within 0–3 months. A subsequent nationwide study in Brazil confirmed these nonlinear and delayed effects, showing that extremely wet conditions increased dengue incidence at short lags, while prolonged drought amplified risk at 3–5 months, particularly in highly urbanized settings [48].

These results parallel those of Kache et al. [46] in Colombia and highlight a recurrent pattern across diverse climatic contexts: intense rainfall may temporarily suppress transmission by flushing breeding sites, but prolonged dry periods can indirectly fuel epidemics by promoting water-storage practices and creating persistent habitats for *Aedes* spp. Critically, this dynamic is further compounded by the chronic water-supply crisis in Santa Marta. According to Londoño et al. [49], the city's water-distribution system is under considerable stress: despite a formal supply-coverage rate of around 91%, the system faces serious deficits because of a combination of severe drought and over-extended infrastructure. During dry periods, the rivers that supply the city drop dramatically in flow, compelling many households to store water in tanks or to connect additional pumps in order to maintain their supply. These conditions provide an ideal environment for vector proliferation, and thus link neatly with the observed lag-effect of decreased rainfall followed by higher dengue incidence.

Temperature effects were also complex. We found that both maximum LSTD and minimum LSTN, at specific lags, were negatively associated with dengue cases. This agrees with recent evidence that extreme heat events may suppress vector survival from research conducted by observing at a temperature of 37 °C [50]. In coastal cities like Santa Marta, high LST can exceed the thermal optimum for *Aedes* mosquitoes, reducing adult survival or egg viability, while moderate conditions a few weeks earlier may be more conducive to transmission. The negative associations observed in our models thus align with the broader literature on nonlinear temperature-dengue relationships [9,51], and with a study carried out in Colombian cities in which the authors found that average and maximum temperatures greater than 28 °C and 32 °C had an inverse association with dengue incidence [18], which is in accordance with our results given that we observed mean numbers of 37 °C and 23 °C for maximum LSTD and minimum LSTN, respectively. Moreover, the positive interaction between NDVI and maximum LSTD indicates that vegetation may partially buffer the negative effects of high temperatures, sustaining dengue transmission under otherwise thermally limiting conditions.

Previous studies in Colombia have consistently documented strong spatial and spatiotemporal structuring of dengue transmission at urban and neighborhood scales. Early analyses in Cali city demonstrated pronounced spatial clustering and temporal persistence of dengue incidence, highlighting the importance of localized transmission processes and neighborhood-level heterogeneity [19]. Subsequent spatial regression studies showed that dengue risk within Cali is shaped by a combination of socioeconomic and environmental factors [20]. Similar space-time clustering has been documented in other hyperendemic Colombian cities such as Girardot [22]. In addition, at broader spatial scales, space-time analyses revealed recurrent and overlapping outbreaks of dengue and other arboviruses across Colombia [21]. More recent work using space-time conditional autoregressive models refined neighborhood-level risk estimation while accounting for spatial and temporal dependence [21]. Complementary time-series studies demonstrated the predictive value of local weather and regional climate variability for dengue dynamics [16].

Our study aligns with this literature in recognizing dengue transmission as a context-dependent process shaped by environmental conditions operating within urban systems. However, it differs in both scale and analytical focus. Rather than emphasizing neighborhood-level spatial clustering or short-term outbreak prediction, this study adopts a long-term, city-scale perspective across 15 years, allowing assessment of how meteorological and environmental drivers operate across interannual variability. Importantly, our results extend previous findings by demonstrating that environmental associations, particularly those related to vegetation greenness, are not uniform but are strongly modulated by population density and thermal conditions. This interaction-based perspective suggests that vegetation enhances dengue risk primarily where dense human populations and favorable microclimates coexist, rather than acting as an independent or universally positive risk factor.

Regarding the limitations of this study, it is essential to note that demographic variables have a limited temporal resolution, being updated approximately every 5–10 years, which limits their use and perhaps their potential in forecasting dengue. Among the limitations of microdata is the possibility of underreporting of data due to the COVID-19 pandemic and the lack of continuity of health personnel. Additionally, we had to work with remote sensing variables, especially in terms of temperature and precipitation, since the meteorological database for Colombia had a lot of missing data. In future studies, it would be interesting to incorporate the “El Niño Oceanic Index” as a sensitive variable to anticipate dengue, since there was a significant relationship between them in a Caribbean region of Colombia [52].

## 5 Conclusion

To sum up, this study provides quantitative evidence of the nonlinear and delayed relationships between dengue risk and meteorological, environmental, and demographic determinants over an extended study period in Santa Marta, Colombia. Previous research supports the idea that dengue transmission is a multifactorial process shaped by the complex interplay of diverse drivers [39]. Climate variables, in particular, are often interdependent and exhibit nonlinear associations. Our results highlight the importance of adopting a One Health approach, where human behaviors (e.g., water storage), environmental changes (e.g., rainfall and vegetation cover), and vector ecology are considered jointly to understand and predict transmission, and offer valuable insights for local health authorities aiming to design early warning systems capable of anticipating future risks. As extreme climate events become more frequent, One Health–informed strategies will be key to building resilient public health responses against mosquito-borne diseases. By demonstrating that environmental drivers of dengue incidence are strongly conditioned by population density, this study provides a clearer understanding of how climatic, environmental, and demographic processes jointly shape transmission risk, thereby strengthening the evidence base for targeted prevention and control strategies in urban settings.

## CRediT authorship contribution statement

**Mia E. Martin:** Writing – original draft, Visualization, Methodology, Formal analysis, Data curation. **Juan A. Insaurralde:** Writing – original draft, Methodology, Formal analysis, Data curation. **Francisco F. Ludueña-Almeida:** Writing – review & editing, Funding acquisition. **Doriam Camacho-Rodríguez:** Writing – original draft, Funding acquisition. **Gabriel Parra-Henao:** Writing – review & editing. **Alexander Salazar-Ceballos:** Writing – original draft, Methodology, Data curation. **Elizabet L. Estallo:** Writing – original draft, Supervision, Funding acquisition, Conceptualization.

## Funding

This study is supported by the InterAmerican Institute of 10.13039/100008086Global Change (IAI) project SG12-CEH.

## Declaration of competing interests

The authors declare the following financial interests/personal relationships which may be considered as potential competing interests:

Elizabet L. Estallo reports financial support was provided by InterAmerican Institute of Global Change (IAI). Doriam Camacho-Rodriguez reports financial support was provided by InterAmerican Institute of Global Change (IAI). If there are other authors, they declare that they have no known competing financial interests or personal relationships that could have appeared to influence the work reported in this paper.
