# Supplementary material for: Assessing environmental and climatic predictors of dengue fever in Santa Marta, Colombia: implications for One Health surveillance
Source: Sci One Health. 2026 Jun 6;5:100164. doi: 10.1016/j.soh.2026.100164 (PMC13315112; doi:10.1016/j.soh.2026.100164)
Supplement: Multimedia component 1 [file mmc1.docx]

**Assessing environmental and climatic predictors of dengue fever in Santa Marta, Colombia: implications for One Health surveillance**

Mia E. Martin, Ariel Insaurralde, Francisco Ludueña-Almeida, Doriam Camacho-Rodríguez, Alexander Salazar-Ceballos, Elizabet L. Estallo

Supplementary Material

**Table S1.** Top 10 generalized linear mixed models (GLMMs) and the null model, selected using the “dredge” function in R (version 4.2.3), ranked by their Akaike information criterion (AIC) values.

| Model | **1** | **2** | 3 | 4 | 5 | 6 | 7 | 8 | 9 | 10 | null |
| --- | --- | --- | --- | --- | --- | --- | --- | --- | --- | --- | --- |
| EVI_max | **x** | **x** | x | x | x | x | x | x | x | x | x |
| EVI_max_3 | **x** | **x** | x | x | x | 0.020 | x | x | x | x | x |
| EVI_sum_1 | **x** | **x** | x | x | x | x | x | x | x | x | x |
| LSTD_max_1 | **−0.234** | **x** | x | −0.239 | −0.195 | −0.248 | −0.313 | −0.338 | −0.248 | −0.341 | x |
| LSTD_min_1 | **x** | **x** | x | x | x | x | 0.115 | 0.137 | x | 0.132 | x |
| LSTD_sd_3 | **x** | **−0.178** | −0.176 | −0.224 | x | −0.186 | −0.193 | -0.219 | −0.187 | −0.221 | x |
| LSTN_max | **x** | **x** | x | x | x | x | x | x | x | x | x |
| LSTN_min_4 | **−0.089** | **−0.104** | −0.089 | −0.069 | −0.093 | −0.094 | −0.085 | x | −0.095 | x | x |
| LSTN_mode | **x** | **x** | x | x | x | x | x | x | 0.020 | 0.020 | x |
| LSTN_mode_3 | **x** | **x** | x | x | x | x | x | -0.020 | x | x | x |
| LSTN_sum_4 | **x** | **x** | 0.089 | x | x | x | 0.112 | x | x | x | x |
| NDVI | **x** | **0.248** | 0.227 | x | 0.204 | x | x | x | x | x | x |
| NDVI_min | **0.132** | **x** | x | x | x | x | x | x | x | x | x |
| NDVI_min_1 | **x** | **x** | x | 0.077 | x | X | x | x | x | x | x |
| NDVI_mode_2 | **x** | **x** | x | x | x | X | x | x | x | x | x |
| NDWI_mode_1 | **−0.137** | **x** | x | −0.119 | x | X | x | -0.149 | x | −0.151 | x |
| popden | **x** | **x** | x | x | x | X | x | x | X | x | x |
| pp_4 | **−0.225** | **−0.172** | −0.176 | x | −0.181 | −0.163 | x | x | −0.160 | x | x |
| pp_min_3 | **x** | **-0.101** | x | x | −0.127 | x | x | x | x | x | x |
| IC | **4942.75** | **4944.05** | 4944.77 | 4944.77 | 4944.93 | 4945.25 | 4945.35 | 4945.36 | 4945.36 | 4945.38 | 5084.000 |
| delta AIC | **0.00** | **1.30** | 2.02 | 2.02 | 2.18 | 2.50 | 2.60 | 2.61 | 2.62 | 2.63 | 141.250 |

Note: The best models are highlighted in bold.
